# Tumor microenvironment enhanced NIR II fluorescence imaging for tumor precise surgery navigation *via* tetrasulfide mesoporous silica-coated Nd-based rare-earth nanocrystals

**DOI:** 10.1016/j.mtbio.2022.100397

**Published:** 2022-08-20

**Authors:** Jiaqi Li, Fukai Zhu, Kangliang Lou, Haina Tian, Qiang Luo, Yongying Dang, Xiaolong Liu, Peiyuan Wang, Liming Wu

**Affiliations:** a The United Innovation of Mengchao Hepatobiliary Technology Key Laboratory of Fujian Province, Mengchao Hepatobiliary Hospital of Fujian Medical University, Fuzhou 350025, PR China; b Key Laboratory of Design and Assembly of Functional Nanostructures, Fujian Institute of Research on the Structure of Matter, Chinese Academy of Sciences, Fuzhou, 350002, PR China; c Department of Hepatobiliary and Pancreatic Surgery, The First Affiliated Hospital, Zhejiang Provincial Key Laboratory of Pancreatic Disease, School of Medicine, Zhejiang University, Hangzhou, 310003, China; d Collaborative Innovation Center of Mushroom Health Industry, Minnan Normal University, Zhangzhou, Fujian, 363000, PR China; e Cancer Center & Department of Breast and Thyroid Surgery, Xiang'an Hospital of Xiamen University, School of Medicine, Xiamen University, Xiamen, 361100, Fujian, China; f Department of Biomaterials, College of Materials, Research Center of Biomedical Engineering of Xiamen & Key Laboratory of Biomedical Engineering of Fujian Province, Xiamen University, Xiamen, 361005, PR China

**Keywords:** Tumor microenvironment response, Down-conversion nanoparticles, NIR II fluorescence Imaging, Signal to background ratio enhancement, Surgery navigation

## Abstract

*In vivo* fluorescent imaging by using the new contrast agents emitted at short-wavelength infrared region (NIR II, 1000–1700 ​nm) presents an unprecedent advantages in imaging sensitivity and spatial resolution over traditional near-infrared (NIR) light. Recently, Nd-based rare-earth nanocrystals have attracted considerable attention due to the high quantum yield (∼40%) of their emission at NIR II. However, undesirable capture by reticuloendothelial system to bring strong background signal is unsatisfying for tumor discrimination. Here, GSH-sensitive tetrasulfide bond incorporated mesoporous silica shell has entrusted onto Nd-based down-conversion nanocrystals (DCNPs) surface to totally quench the fluorescence of DCNPs. After RGD conjugation on the silica surface, the NIR II contrast agents could actively target to liver tumors. Then tetrasulfide bonds can be broken during the silica framework decomposing in cytoplasm under high GSH concentration to result in NIR II fluorescence explosive recover. Benefiting from this specific response under tumor microenvironment, the NIR II signal in other organs was markedly reduced, while the signal-to-background ratio is prominently enhanced in tumors. Then, solid liver tumors were successfully resected under the guidance of our GSH responsive NIR II fluorescent imaging with no recurrence after 20-day of surgery. Meanwhile, by combining with the ignorable side effects, the Nd-based nanoprobes vastly improved the imaging resolution of tumor margin, opening a paradigm of NIR II fluorescent imaging-guided surgery.

## Graphical abstract

**Figure:**
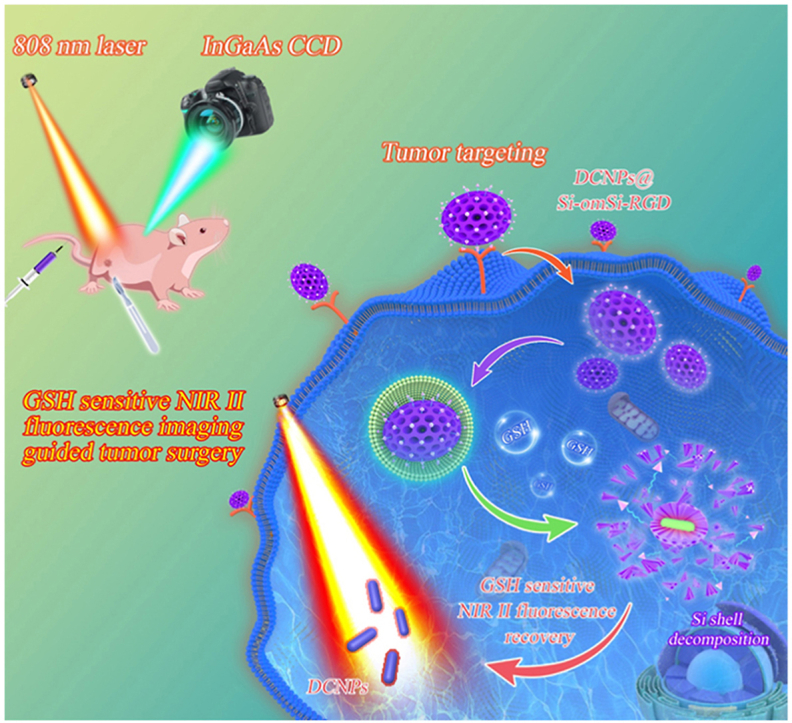

## Credit author statement

Liming Wu: Study design and supervision. Peiyuan Wang: Study design, Supervision and writing – reviewing and editing. Xiaolong Liu: Study design, Supervision, founding and resource. Jiaqi Li: Material preparation, characterization, biological examination, animal related experiments, Data curation, Writing – original draft. Fukai Zhu: Material preparation, characterization, biological examination, writing-supplement. Kangliang Lou: Animal related experiments. Yongying Dang: Characterization. Haina Tian: Animal related experiments.

## 1 Introduction

Hepatocellular carcinoma is one of the most common malignancies worldwide and it ranks the fourth leading cause of tumor-related mortality [[1], [2], [3]]. Surgical resection has been performed to combat solid tumors in combining with other therapeutic modalities, like chemotherapy, radiotherapy and immunotherapy, *etc*. Intraoperative visualization of the contour profile of malignant tumors has significant implications for the surgery outcomes. Nevertheless, clinical surgeons often mainly rely on visual feedback or experience to identify tumor margins from health tissues that often caveats of leaving tumor tissue residue or dissecting health tissue excessively [4,5]. Hereby, intraoperative diagnostics of residual tumor tissue still remains challenge. The clinical common used imaging techniques such as Magnetic Resonance Imaging (MRI) [6], Ultrasound Imaging (US) [7], Positron Emission Tomography (PET) [8] and Computed X-ray tomography (CT) [9], *etc.* are mostly preferable for preoperative tumor detection. Due to sensitivity and specificity limitation, long time acquisition as well as ionizing radiation risk that above imaging modalities are not applicable to intraoperative navigation. Fortunately, *in vivo* NIR fluorescence imaging has developed as a non-invasive tool for enhancing staging tumor diagnosis, especially, it has unique advantages in guiding tumor resection due to its high-spatial-resolution and instantaneity [[10], [11], [12], [13]]. The US Food and Drug Administration (FDA) has approved clinical useable contrast agents in the first near-infrared window (NIR I, 750–900 ​nm), like indocyanine green (ICG) [14] and methylene blue (MB) [15] which have been extensively explored for variety tumor margin delineation; however, short time of tumor retention, photo bleaching and rapid systematic clearance have impended these fluorophores for fluorescence imaging guided tumor surgery *in vivo* [16]. Moreover, the penetration depth limitation (∼3 ​mm) and high autofluorescence from background to normal tissues of these clinical commonly used organic probes have substantially placed restrictions on the reflectance-based intraoperative imaging [17,18]. The second near-infrared region (NIR Ⅱ, 1000–1700 ​nm) fluorescent imaging is promising for intraoperative tumor imaging due to high resolution of sub-10 μm with penetration depth of several centimeters and low background signals, which are satisfying for both preoperative diagnosis and intraoperative surgery navigation.

Commonly used NIR-II probes include quantum dots (QDs) [19], organic dyes [20], single-walled carbon nanotubes (SWCNTs) [21], were often unsuitable for long-term *in vivo* fluorescent imaging, on accounting of inherent toxicity, short blood half-lives or poor biocompatibility. Furthermore, they also face challenges of photo-bleaching and reticuloendothelial system (RES) capture for the widespread adoption in clinical [[22], [23], [24]]. Interestingly, rare-earth doped down-conversion nanoparticles (DCNPs) with core-shell nanostructure, especially Nd-based nanocrystals, not only had high quantum efficiency (QY: ∼40%) [25,26] and stable photo-stability, but also with a long blood half-life and low off-target side effect *in vivo,* present as ideal NIR II contrast agents for intraoperative tumor surgery navigation in preclinical and clinical trials [[27], [28], [29], [30], [31]]. The development of tumor microenvironment responsive intelligent nanoprobes have attracted increasing interesting to enhance the signal to back ground ratio (SBR) for malignant tumor discrimination [32]. However, imaging guided tumor resection surgery with tumor microenvironment activated Nd-based nanoprobes has little been reported thus far. Silica coating could make core nanoparticles water-dispersible, biocompatible and versatile surface modification. Among the most ideal nanostructures, mesoporous silica nanomaterial (MSN) has been approved for biomedical application by FDA [33]. Meanwhile, in order to construct an intelligent property, as a cleavable active cross-linking, tetrasulfide bond which is capable of be incorporated into the mesopore framework of MSN has attracted tremendous attention on accounting of its unprecedented biocompatibility and tumor microenvironment responsive capabilities [34]. Previous studies have demonstrated that tetrasulfide-bridged MSNs often present superior structural stability under physiological buffers, while exhibits rapidly biodegradable performance following redox stimulation of high GSH microenvironment [[35], [36], [37], [38]]. Importantly intracellular GSH concentration is 100–1000 times higher than that of extracellular fluid in tumor site [[39], [40], [41], [42]]. Therefore, tetrasulfide bond co-doped NIR II fluorescent contrast agent seems a promising candidate for SBR enhancement during intraoperative fluorescent imaging.

**Fig. 1:**
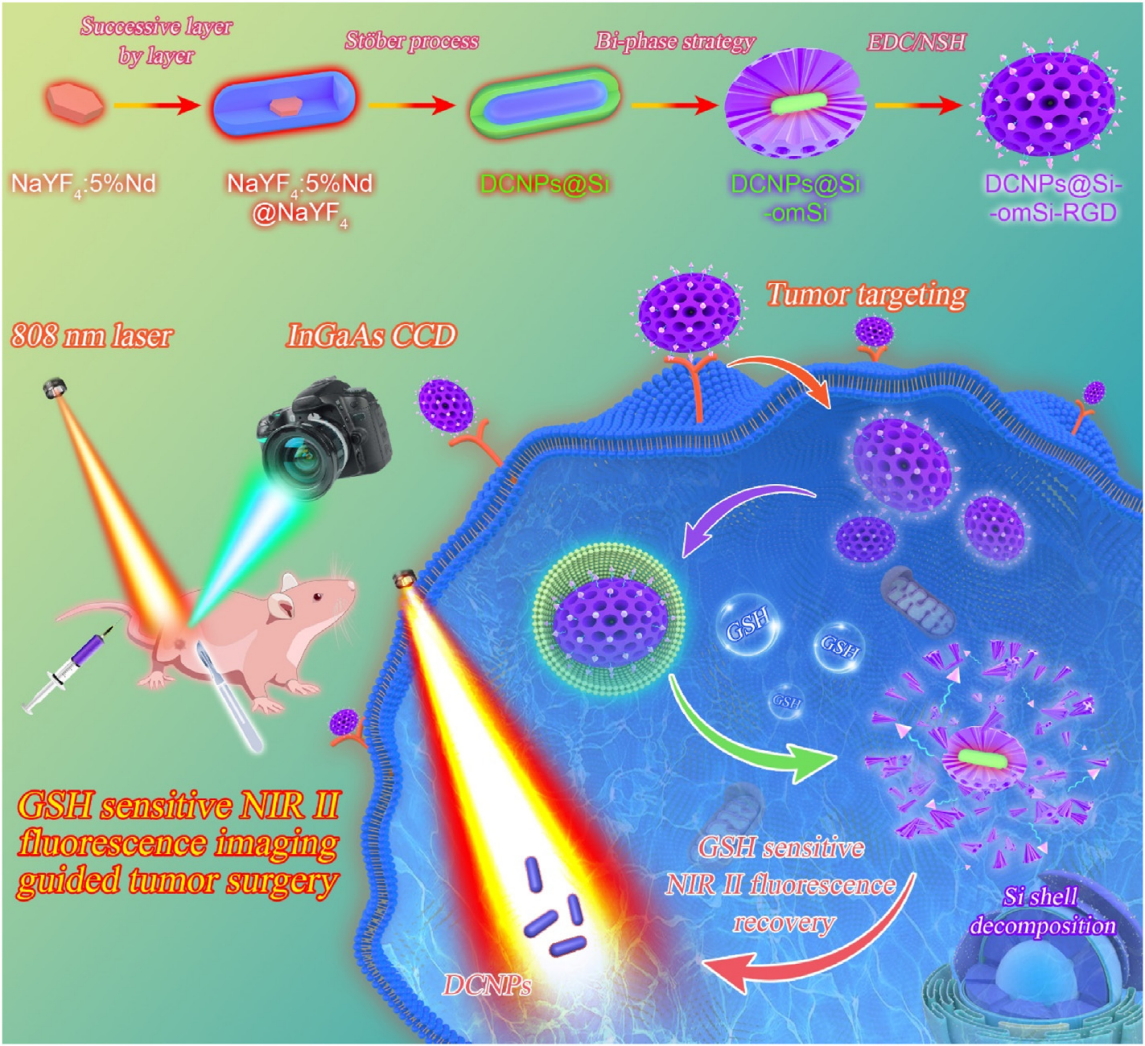
GSH sensitive DCNPs@Si-omSi-RGD fabrication with signal to background ratio enhacement for liver tumor precise resection under NIR II fluorescence imaging guidance.

For above reasons, in this study, Nd-based core-shell DCNPs, NaYF_4_:5%Nd@NaYF_4_ nanocrystals were constructed with unique optical properties of 808 ​nm excitation and 1064 ​nm fluorescent emission. In order to transfer this hydrophobic DCNPs into hydrophilic stage, solid silica shell was coated on lanthanide-based nanocrystals (DCNPs@Si). Subsequently, a dendritic out layer, GSH responsive organo-mesoporous silica was fully and homogeneously wrapped on the solid silica surface (DCNPs@Si-omSi). Owing to light-scattering effect on both incident and emissive lights, NIR II fluorescence from DCNPs could be efficiently diminished. After hepatic targeting peptide RGD modification (DCNPs@Si-omSi-RGD), this unique NIR II nanoprobes could specifically accumulated in liver tumors after intravenous injection, afterwards the tetrasulfide mesoporous silicon could be degraded in cytoplasm with high concentration of GSH to recovery the NIR II fluorescence, realizing the purpose of SBR improvement in tumor tissue. Tumors were then resected at maximum SBR timepoint under NIR II fluorescent imaging guidance. According to histological staining results, the DCNPs@Si-omSi-RGD can successfully discriminate the tumor outline (Fig. 1). Combine with low off-targeting side effects, the GSH sensitive NIR II nanoprobes had the advantages of real-time imaging and accurate tumor resection under 808 ​nm laser irradiation. Above findings favor the clinic applications of the advanced NIR II fluorescent imaging method and open up the new possibility of Nd-based contrast agents for clinical malignant tumor dissection.

## 2 Experiments

### 2.1 Preparation of DCNPs@Si-omSi

DCNPs@Si was firstly synthesized as follows. 7 ​mL *n*-octanol, 12 ​mL triton X-100 and 50 ​mL cyclohexane were added into a 100 ​mL bottle. Then the mixture was stirred for 30 ​min under room temperature. 2 ​mL as-prepared DCNPs were added and stirred at room temperature for 45 ​min. Then 0.45 ​mL of ammonia and 0.3 ​mL TEOS were slowly added and stirred for another 45 ​min to obtain a microemulsion solution. Finally, the above reaction mixture was stirred at room temperature for 18 ​h. Finally, the as-prepared sample was washed with anhydrous ethanol and water for three times. The final NaYF_4_: 5% Nd@NaYF_4_@Si product was dispersed in 20 ​mL DI water.

Subsequently, organo-mesoporous silica shell was coated on silica shell. Firstly, 5 ​mL DCNPs@Si prepared in the previous step and 25 ​mL DI water were added into the round-bottom flask, and then 1.5 ​g hexadecyltrimethylammonium bromide (CTAB) was added, afterwards the above solution was mixed together by ultrasound. Then 0.75 ​mL 0.25 ​*wt* % triethanolamine (TEA) was added into above aqueous solution at the oil bath under 60 ​°C. After 300 ​rpm stirred for 10 ​min, a silica precursor contained 5 ​mL cyclohexane, 0.05 ​mL bis[3-(triethoxysily)propyl] tetrasulfide (BTES) and 0.2 ​mL tetraethyl orthosilicate (TEOS) was added slowly. The reaction was performed at 60 ​°C oil bath for 24h. Finally, the prepared production was washed with anhydrous ethanol and DI water for three times, respectively. The DCNPs@Si-omSi was redispersed in 10 ​mL DI water.

### 2.2 NIR II Fluorescence stability

Firstly, the DCNPs@Si-omSi was dissolved in water, 10% FBS and 0.9% NaCl at equivalent amounts, respectively. The ICG dissolved in the above three solvents with the same fluorescence intensity was prepared in 0.5 ​mL centrifuge tubes. Then all tubes were irradiated by 808 ​nm laser for 30 ​min. NIR II fluorescent images were taken every minute to record the fluctuation of fluorescence intensity. Secondly, DCNPs@Si-omSi and ICG dissolved in above various solutions were also prepared in 96-well plates and all of them were placed at room temperature under day light illumination. NIR II fluorescent images were obtained 0, 1, 3, 6, 12, 24, 36, 48, 72 and 96 ​h under day light irradiation.

### 2.3 GSH triggered biodegradation of DCNPs@Si-omSi-RGD

Firstly, 10 ​mM GSH solution was prepared, then 100 μg/mL DCNPs@Si-omSi was mixed with 5 ​mL GSH solution. Afterwards, the mixture was putted at shaking table at 37 ​°C for different period (1 ​h, 6 ​h, 12 ​h, 24 ​h, 36 ​h, 48 ​h and 72 ​h). Latterly, the degraded DCNPs@Si-omSi was collected and washed by water. Finally, all samples were observed under TEM.

### 2.4 Cellular uptake evaluation via flow cytometry

Herein, as a commercial probe, the FITC was anchored on the surface of DCNPs@Si-mSi to study the endocytosis by HepG2 cells. 2 ​× ​10^5^ tumor cells were firstly seeded in a 12-well plate for 12 ​h incubation at DMEM medium (10% FBS, 100 units/mL of penicillin and 100 ​μg/mL of streptomycin) and the cell coverage could be reached to 90%. Then FITC (equal FITC dose as DCNPs@Si-omSi-RGD@FITC), DCNPs@Si-omSi@FITC, DCNPs@Si-omSi-RGD@FITC and RGD ​+ ​DCNPs@Si-omSi-RGD@FITC at a concentration of 30 ​μg/mL were added for 1 ​h, 2 ​h, 4 ​h, 8 ​h, 10 ​h and 12 ​h incubation, respectively. The cells were digested and resuspended with PBS, finally the intracellular FITC intensity was analyzed by flow cytometry.

### 2.5 CLSM studies of cellular uptake

Briefly, HepG2 cells were dispersed in the 12-well plate and incubated for 12 ​h with 1 ​mL of DMEM (10% FBS, 100 units/mL of penicillin and 100 ​μg/mL of streptomycin). After the treatment with DCNPs@Si-omSi@FITC and DCNPs@Si-omSi-RGD@FITC at a concentration of 100 ​μg/mL, the liver tumor cells were incubated for 1 ​h, 2 ​h, 4 ​h, 8 ​h, 10 ​h and 12 ​h, respectively. Furthermore, the HepG2 cells were treated with free FITC (equal FITC dose as DCNPs@Si-omSi-RGD@FITC), PBS, DCNPs@Si-omSi@FITC, DCNPs@Si-omSi-RGD@FITC and RGD ​+ ​DCNPs@Si-omSi-RGD@FITC tfor 8 ​h. Then, all the samples were fixed with formalin for 20 ​min. Finally, all cells were washed with 1X PBS for three times, and then a moderation amount of DAPI (1 ​μg/mL in PBS) was applied to stain the nuclei for 20 ​min prior for further observation under confocal laser scanning microscope (CLSM, Nikon A1RMP imaging system).

### 2.6 Subcutaneous liver tumor model construction

All *in vivo* studies were received permission from the animal ethics committee of Mengchao Hepatobiliary Hospital of Fujian Medical University. All studies were performed in accordance with relevant guidelines. 6-8-week-old Balb/c mice were brought from Shanghai SLAC Laboratory Animal Co., Ltd., luciferase labeled HepG2 (HepG2-luc) cells were cultured for 24 ​h under DMEM ((10% FBS, 100 units/mL of penicillin and 100 ​μg/mL of streptomycin). Then liver tumor cells were collected and washed by sterilized 1 ​× ​PBS. Balb/c female mice were received intramuscular injection of HepG2-luc cells at right hind limb. The tumor volume was calculated by a formula of (width ​× ​width ​× ​length)/2. It could grow into 200–300 ​mm^3^ after postinjection of tumor cells at the day 14. NIR II fluorescent imaging guided tumor resection were performed at this tumor size.

### 2.7 NIR II fluorescence imaging guided tumor surgery

Subcutaneous liver tumor model mice were randomly divided into bright light surgery group and NIR II fluorescence surgery group (n ​= ​5). In order to confirm the subcutaneous liver tumor construction, bioluminescence imaging was performed in HepG2-luc tumor bearing mice by IVIS-II imaging system. Then mice were intravenously injected with DCNPs@Si-omSi-RGD and NIR II fluorescent imaging was operated under 808 ​nm laser illumination (200 ​mW/cm^2^, 1000 ​nm long-pass filter) at various timepoints of post-injection. SBR reached to the maximum level at 48 ​h post-injection, the contour of the tumor could be successfully determined by NIR II fluorescence imaging, therefore, the main tumor body was removed during the first resection (first cut). Then the mice were still placed under the NIR-II fluorescence imaging system for residual tumor tissue determination. Subsequently, tissue with residual fluorescence signal was further removed during the second resection (second cut). Simultaneously, the muscle tissue of peritumoral tumor was also collected. Finally, *ex vivo* NIR II fluorescence imaging of two resected tumor tissue and muscle tissue was also performed; furthermore, the H&E pathological analysis of above three tissues was conducted. The body weight and survival rate of the mice were monitored after surgery for 28 days. Meanwhile, tumors were removed under the bright light and bioluminescence imaging of both two groups after tumor tissue resection at the 1st day and the 20th day *via* IVIS-II imaging to determine whether the tumor had metastasis or recurrence.

### 2.8 Pathological analysis of toxicity *in vivo*

The Balb/c mice were received tail vein injection of DCNPs@Si-omSi-RGD, and NIR II fluorescent imaging guided tumor surgery at 48 ​h post-injection was subsequently carried out. Then they were randomly divided into five groups: 1-day, 3-day, 7-day and 28-day post-surgery, respectively. Mice with intravenous injection of PBS for 1 day was set as the control group. Blood routine and biochemical indexes were analysis in all groups. All mice were sacrificed and the main organs (heart, liver, spleen, lung, renal and brain) were harvested for H&E staining. At the same time, the acute toxicity and subcutaneously inflammation studies were conducted in healthy Balb/c mice. They were sacrificed at the 1st day, the 7th day and the 28th day after intramuscularly injection with 5 ​mg/kg DCNPs@Si-omSi-RGD. Mice with intramuscularly injection of PBS for 1 day was set as the control. Finally, the subcutaneous muscle tissues were collected for further H&E staining analysis.

### 2.9 Statistical analysis

All data are presented as mean value ​± ​standard deviation. The statistical analysis was conducted by the software of SPSS 20.0 and ∗∗ represented p ​< ​0.05, which was referred to statistically significant. For the cellular uptake comparison analysis, one-way ANOVA followed by Tukey post hoc analyzation was used after the various treatments (n ​= ​5).

## 3 Results and discussion

### 3.1 Stepwise construction of DCNPs@Si-omSi-RGD

**Fig. 2:**
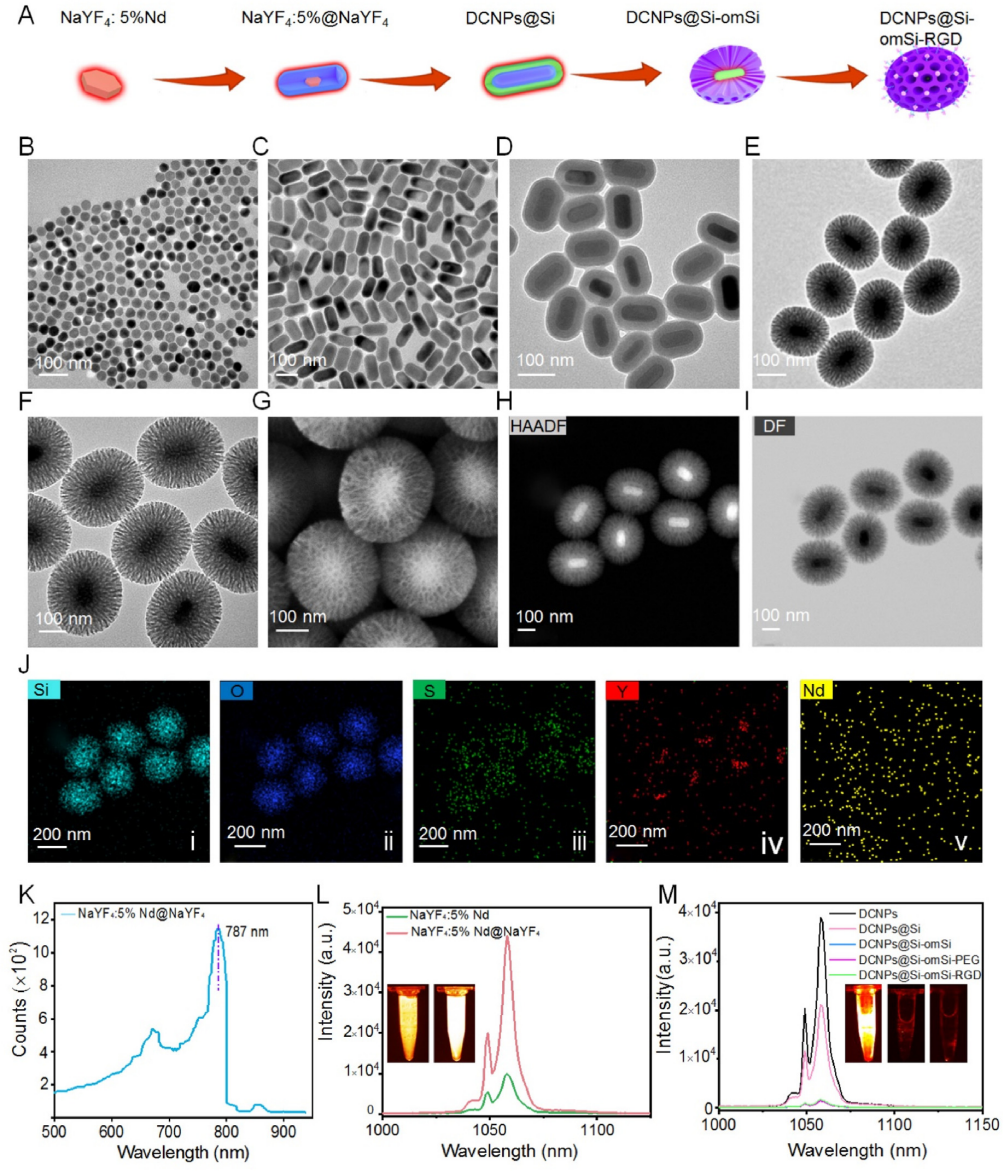
(A) Schematic illustration of stepwise DCNPs@Si-omSi-RGD fabrication. TEM image of (B) NaYF_4_:5%Nd, (C) *β*-phase NaYF_4_:5%Nd@NaYF_4_, (D) DCNPs@Si, (E) DCNPs@Si-omSi and (F) DCNPs@Si-omSi-RGD, respectively. (G) SEM, (H) HAADF and (I) STEM-dark field images of DCNPs@Si-omSi. (J) Elemental mapping images of DCNPs@Si-omSi and the corresponding Si (i), O (ii), S (iii), Y (iv) and Nd (v) distribution, respectively. (K) Excitation spectrum of *β*-phase NaYF_4_:5%Nd@NaYF_4_. (L) Fluorescence spectra of NaYF_4_:5%Nd and *β*-phase NaYF_4_:5%Nd@NaYF_4_. Insert, from left to right, NIR II fluorescent images of OA capped NaYF_4_:5%Nd and OA capped *β*-phase NaYF_4_:5%Nd@NaYF_4_. (M) Fluorescence spectra of OA capped DCNPs, DCNPs@Si, DCNPs@Si-omSi, DCNPs@Si-omSi-PEG and DCNPs@Si-omSi-RGD. Insert, from left to right, NIR II fluorescent images of DCNPs@Si, DCNPs@Si-omSi and DCNPs@Si-omSi-RGD.S

Firstly, ∼25 ​nm NaYF_4_:5%Nd core was primarily prepared under solvothermal reaction at argon atmosphere (Fig. 2A, **B**). The core nanocrystals with monodispersity and uniformed size inspired us to coat a NaYF_4_ layer to passivate core surface defects and simultaneously insulate from the local environment. Two phases of Nd-based core-shell DCNPs (Fig. 2C, **S1**) could be successfully fabricated according to the previously reported successive layer-by-layer growth method [43]. ∼75 ​nm *α-*phase can transform into ∼100 ​nm *β*-phase after increasing the temperature from 280 ​°C to 300 ​°C during NaYF_4_ shell wrapping on NaYF4:5%Nd nanocore. The spectrum data of *β*-phase illustrated that visible-infrared absorbance spectrum with a strong peak at ∼787 ​nm (Fig. 2K), therefore, 808 ​nm laser was employed for excitation the photoluminescence of Nd-based nanocrystals. We found that NIR II fluorescence intensity was significantly enhanced after NaYF_4_ coating under 808 ​nm excitation (Fig. 2L). Interestingly, the fluorescence intensity at 1064 ​nm at *β*-phase was distinctly stronger than that of *α*-phase under 808 ​nm laser excitation (Fig. S2). Due to *β*-phase possesses more excellent photoluminescence quantum yield than the *α*-phase [44], so *β*-phase DCNPs were chosen for the following investigation. Then the preparation details of DCNPs@Si-omSi-RGD are provided as follows. As a versatile surface modification, solid silica coating could make core nanoparticles water-dispersible and biocompatible [45,46]. Therefore, a layer of solid silica with ∼15.5 ​nm thickness was firstly successfully coated on the surface of DCNPs *via* a typical Stöber method (denote as DCNPs@Si) (Fig. 2D) with a reverse-micelle mixture containing CO-520, anomia and cyclohexane [47]. Subsequently, mesoporous organo-silica out-shell with tetrasulfide co-doping was further coated on the as-prepared DCNPs@Si (DCNPs@ Si-omSi) *via* a reported bi-phase approach [48] with CTAB as the template and 25% TEA as the reducing agent (Fig. 2E). Dendritic mesopores (∼60 ​nm) of DCNPs@Si-omSi could be clearly observed under both TEM and Scanning Transmission Electron Microscopy (SEM) images (Fig. 2E, **G**). Size distribution was determined by dynamic scattering analysis and it gradually increased from ∼25 ​nm to ∼255 ​nm after three-layer coating on core nanocrystals, further indicating that DCNPs, DCNPs@Si and DCNPs@Si-omSi were successfully constructed step-by-step (Fig. S3). Interesting, we found the thin layer solid silica layer was detected in DCNPs@Si-omSi (Fig. 2E, **G**). We speculated that tetrasulfide silica precursor, BTES may affected the pH value in water/oil interface, and the vast bulk of solid silica would undergo etching without the protection of CTAB on the surface [49]. Then high angle annular dark field STEM (HAADF-STEM) was utilized to analyze all elements distribution in DCNPs@Si-omSi. The thin wall of solid silica could be quite distinctly observed in both HAADF and dark field images (Fig. 2H, **I**). All images displayed that both Y and Nd elements were found in DCNPs. Simultaneously, O, Si, and S were distributed in mesoporous shell, further validating that hollowed GSH sensitive mesoporous silica was successfully coated on DCNPs (Fig. 2J). Finally, zeta potential of DCNPs@Si-omSi changed from negative value to positive value after DSPE-PEG_2000_-NH_2_ functionalization, revealing the successful modification of amino groups on Si-omSi shell (Fig. S4). Then the RGD molecules were conjugated on DCNPs@Si-omSi *via* EDC/NHS reaction (DCNPs@Si-omSi-RGD). A signal broad peak adsorption was found in UV–*vis* spectrum of DCNPs@Si-omSi-RGD, which is the characteristic absorption of RGD (Fig. 2F, **S5**). According to FTIR analysis, the peak of 1070 ​cm^−1^ was attributed to -C-O- in silica shell and it remarkably attenuated in DCNPs@Si-omSi-RGD (Fig. S6). All spectra data demonstrated the successfully anchoring of RGD in omSi shell. Fig. 2M shows the NIR II emission spectrum of DCNPs@Si-omSi-RGD dispersed in aqueous solution under 808 ​nm laser irradiation. In sharp contrast, the intensity at 1064 ​nm is significantly suppressed in both DCNPs@Si-omSi and DCNPs@Si-omSi-RGD. Because of the silica layer could substantially attenuate the fluorescence of lanthanide nanocrystals [50,51], in this work, we showed that the NIR II fluorescence can be prevented completely by ​∼ ​72 ​nm silica coating.

### 3.2 Characterization of material stability and degradation

**Fig. 3:**
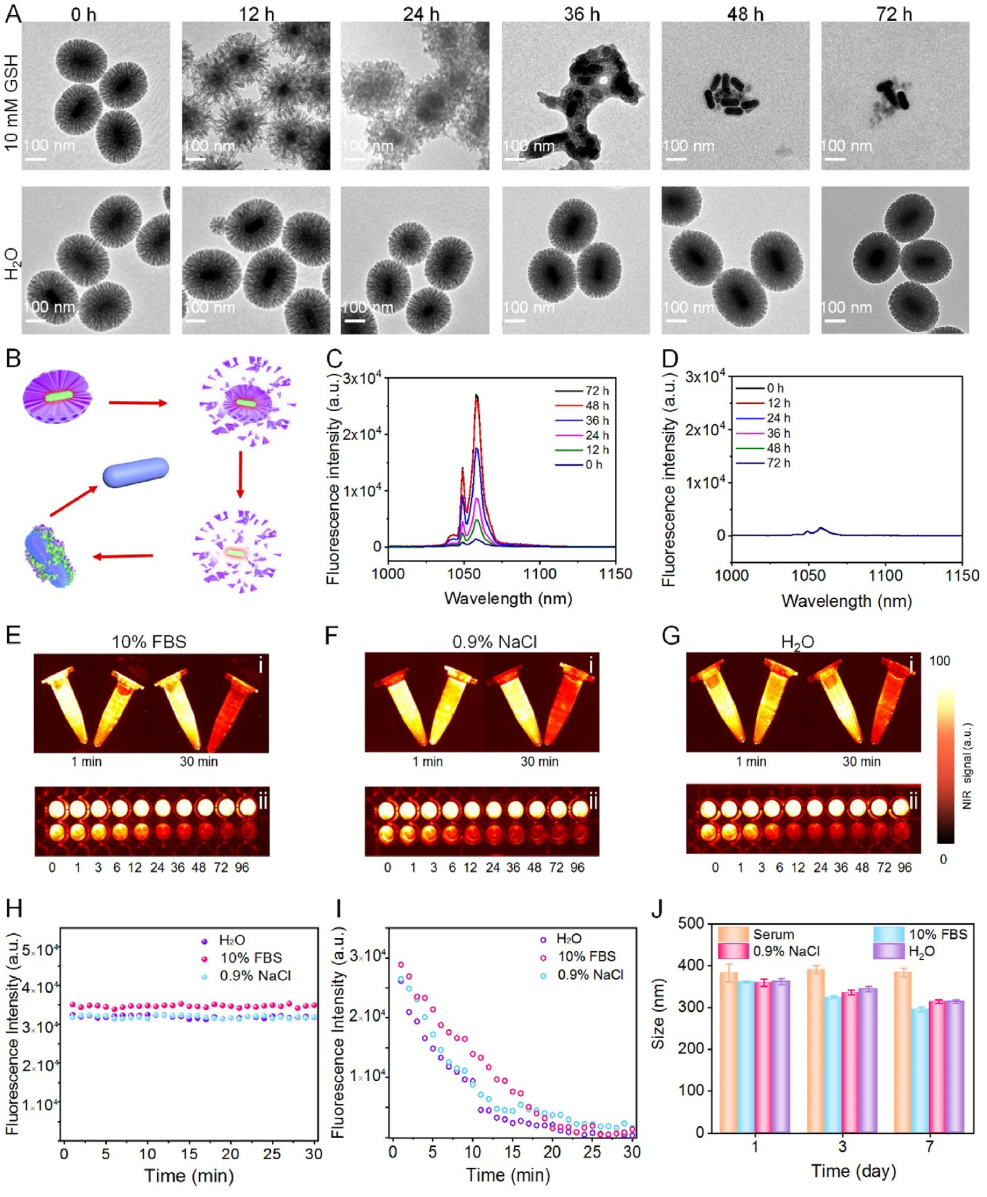
(A) TEM images of DCNPs@Si-omSi after incubated with 10 ​mM GSH buffers and H_2_O for 0 ​h, 6 ​h, 12 ​h, 24 ​h, 36 ​h, 48 ​h and 72 ​h, respectively. (B) Schematic illustration of DCNPs@Si-omSi degradation under 10 ​mM GSH incubation. The fluorescence spectra of degraded DCNPs@Si-omSi in the solution of 10 ​mM GSH (C) and H_2_O (D) for different durations. NIR II fluorescent images of DCNPs@Si-omSi and free ICG dispersed in 10% FBS (Ei), 0.9% NaCl (Fi) or H_2_O (Gi) under continuous 808 ​nm laser irradiation for 30 ​min. NIR II fluorescent images of DCNPs@Si-omSi and free ICG dispersed in 10% FBS (Eii), 0.9% NaCl (Fii) or H_2_O (Gii) in daylight for different hours. Photostability of DCNPs@Si-omSi (H) and free ICG (I) in various biological buffers under continuous 808 ​nm laser illumination. All DCNPs@Si-omSi was pretreated with 10 ​mM GSH for 48 ​h pretreatment in E-H. (J) Size distribution of DCNPs@Si-omSi in a variety of biological solutions for 7 days.

In order to characterize the degradation behavior of tetrasulfide bond in the mesoporous framework toward redox condition, DCNPs@Si-omSi was added into 10 ​mM GSH buffer for various incubations. TEM images (Fig. 3A) displayed that at 12 ​h, the mesoporous shell became sparser and thinner. From 24 ​h on, GSH sensitive mesoporous shell underwent rapid degradation with mesoporous framework disappeared and the thin solid silica shell also became looser, particularly, at 48 ​h, the nanostructure of two silica shells disintegrated into small fragments with only bare DCNPs can be observed. Analogous to 48 ​h, almost all silica skeleton collapsed after 72 ​h. The silica precursor ratio of BTES and TEOS for omSi shell coating was set as 1:4, which was significantly higher than the reported hollowed organo-mesoporous silica [52], resulting in rapid biodegradation under GSH triggering*.* Even though the NIR II fluorescence of DCNPs was partially quenched after solid silica coating (Fig. 2M), it totally degraded at this time point revealing that the solid silica layer had little effect on NIR II fluorescence recovering*.* In contrast, the mesoporous shell with sulfur containing exhibited no discernable structural breakdown when co-incubated with H_2_O for 72 ​h (Fig. 3A, **B**). Attractively, the fluorescence of DCNPs@Si-omSi gradually recovered with the extension of degradation time, and there was an obvious transition at 48 ​h (Fig. 3C), however, the fluorescent of pure water incubated group presented indiscernible changes, which is consisted with TEM results (Fig. 3D). All data preliminarily proved that the tetrasulfide bond co-doped mesoporous shell could be utilized for GSH responsive NIR II fluorescence recovery. Furthermore, our Nd-based nanoprobes showed gradual NIR II fluorescence enhancement under tumor microenvironment, while ignorable fluorescent intensity could be detected in normal tissue. This fascinating tumor microenvironment responsive property could realize significant SBR improvement during malignant and healthy tissue distinguishing *in vivo*. Currently, there are still no commercially available and clinically approved NIR II fluorophores; although ICG can act as a NIR II fluorophore with fluorescent emission tails extending into the NIR II window, its fluorescent signal will be quickly bleached under 808 ​nm laser irradiation, which is not suitable for long-time surgical navigation [53,54]. In order to investigate the photostability of our NIR II nanoprobe, both ICG and degraded DCNPs@Si-omSi (48 ​h 10 ​mM GSH pre-treatment) were incubated at various biological buffers, like 10% FBS, 0.9 ​*wt*% NaCl and pure water, respectively. According to the NIR II fluorescent images, lanthanide-based nanocrystals present stable signals at diverse buffers even after continuously 808 ​nm laser irradiated for 30 ​min, on the contrary, all ICG groups were practically bleached (Fig. 3Ei, **Fi**, **Gi**). Consistent with the fluorescent intensity values (1064 ​nm) detected from at various media, the maximum emission peak value (840 ​nm) of ICG drastically dropped, especially in H_2_O, with almost 92% fluorescent intensity bleached after a 808 ​nm continuous-wavelength laser exposure for 20 ​min (Fig. 3H, **I**). Meanwhile, in the condition of natural light, 100% of the initial fluorescence signal in degraded DCNPs@Si-omSi group was totally maintained over 96 ​h incubation; in sharp contrast, 95% fluorescence signals of ICG were totally quenched only after 24 ​h incubation (Fig. 3Eii, **Fii**, **Gii**). All data proved superior photostability of Nd-based nanoprobes in comparison with free ICG, particularly for time-lapse irradiation of surgical operation with long period. Similarly, stable size distribution was discovered in DCNPs@Si-omSi under different buffers (serum, 10% FBS and 0.9% NaCl) for 7 days, suggesting the biostability in normal tissue during NIR II fluorescent imaging (Fig. 3J). These results demonstrated the overwhelming advantages of DCNPs@Si-omSi for precise tumor surgery under fluorescence imaging guidance.

### 3.3 Cytotoxicity and cellular uptake investigation

**Fig. 4:**
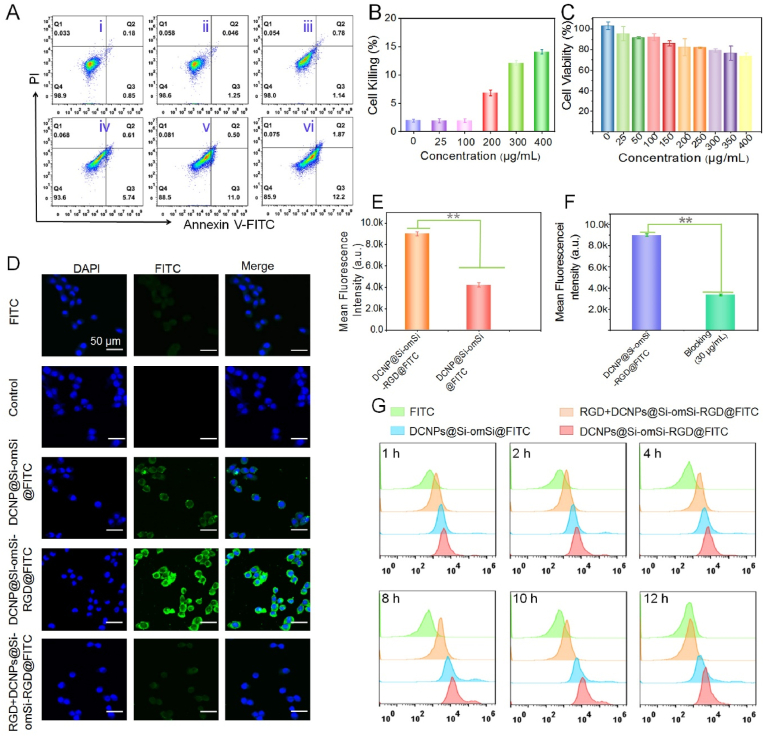
(A) Cell apoptosis/necrosis analysis of HepG2 cells after incubated with different concentrations of DCNPs@Si-omSi-RGD@FITC for 12 ​h, i to vi corresponding to PBS, 25 ​μg/mL, 100 ​μg/mL, 200 ​μg/mL, 300 ​μg/mL, 400 ​μg/mL respectively. (B) Quantitative analysis of cell apoptosis/necrosis in A. (C) The cytotoxicity effect of HepG2 cells after incubated with DCNPs@Si-omSi-RGD for 12 ​h. (D) CLSM images of HepG2 cells after incubated with PBS, free FITC, DCNPs@Si-omSi@FITC, DCNPs@Si-omSi-RGD@FITC and DCNPs@Si-omSi-RGD@FITC with RGD pretreatment for 8 ​h. (E) Quantitative analysis of mean fluorescence intensity among DCNPs@Si-omSi and DCNPs@Si-omSi-RGD, DCNPs@Si-omSi-RGD and RGD ​+ ​DCNPs@Si-omSi-RGD (F) in D. (G) Flow cytometry analysis of green fluorescence in HepG2 cells corresponding to with PBS, free FITC, DCNPs@Si-omSi@FITC, and DCNPs@Si-omSi-RGD@FITC co-culture for different times.

Before *in vivo* fluorescent imaging investigation, the potential cytotoxicity of DCNPs@Si-omSi-RGD was determined in a human liver carcinoma cell line HepG2. As demonstrated in Fig. 4A and B, only 14.1% of cell apoptosis/necrosis was found after 12 ​h incubation with 400 ​μg/mL Nd-based nanoprobes by Annexin V-FITC/PI assay analysis, demonstrating good biocompatibility of the lanthanide nanoprobes. To fully study the cell viability of DCNPs@Si-omSi-RGD against HepG2, we then performed the commonly used MTT assay. The cells exhibited over 80% viability after treatment with 400 ​μg/mL DCNPs@Si-omSi-RGD for 12 ​h, proving that NIR II nanoprobe essentially did not affect cell proliferation, making it suitable for subsequent therapeutic applications (Fig. 4C), although there was certain increase of ROS level after 400 ​μg/mL DCNPs@Si-omSi-RGD incubation (Fig. S7). Encouraged by above excellent biosafety, the cellular targeting capability of DCNPs@Si-omSi-RGD was evaluated by CLSM after FITC loading in the mesoporous silica shell. Specifically, we observed that intracellular green fluorescence in DCNPs@Si-omSi-RGD@FITC group was remarkedly higher than free FITC, PBS and DCNPs@Si-omSi@FITC groups, demonstrating the active tumor targeting performance of RGD toward the α_ν_β_3_ receptor in tumor cell membrane (Fig. 4D). Additionally, integrin blocking experiment was also assessed after free RGD pre-treatment. Crucially, ignorable fluorescence signal was found when the cells were subsequently co-cultured with DCNPs@Si-omSi-RGD@FITC. Quantitatively analysis of green signals in DCNPs@Si-omSi-RGD@FITC, DCNPs@Si-omSi@FITC and free RGD ​+ ​DCNPs@Si-omSi-RGD@FITC groups intuitively displayed that RGD modification could efficiently enhance the active targeting toward tumor cells (Fig. 4E, **F**). Immediately, we also performed flow cytometry analysis and found that the intensity of all groups steadily increased with maximum endocytosis at 8 ​h and those cells treated with DCNPs@Si-omSi-RGD@FITC showed stronger fluorescence in comparison with other groups at all incubation time-points, suggesting the occurrence of active cell targeting in the presence of nanoprobes with RGD modification (Fig. 4G). At the same time, CLSM images of HepG2 cells incubated with DCNPs@Si-omSi@FITC and DCNPs@Si-omSi-RGD@FITC were then studied as a function of incubation time. Comparing with DCNPs@Si-omSi@FITC, the DCNPs@Si-omSi-RGD@FITC was found to be indeed more efficient in cellular uptake (Fig. S8-**S9**). Obviously, the green signal inside cells significantly enhanced after 8 ​h incubation, in accordance with flow cytometry results (Fig. S10). Therefore, we can expect that our nanoprobe would realize the prominent tumor targeting effect.

### 3.4 Tumor targeting and NIR II fluorescence imaging guided tumor surgery in liver tumor bearing mice

**Fig. 5:**
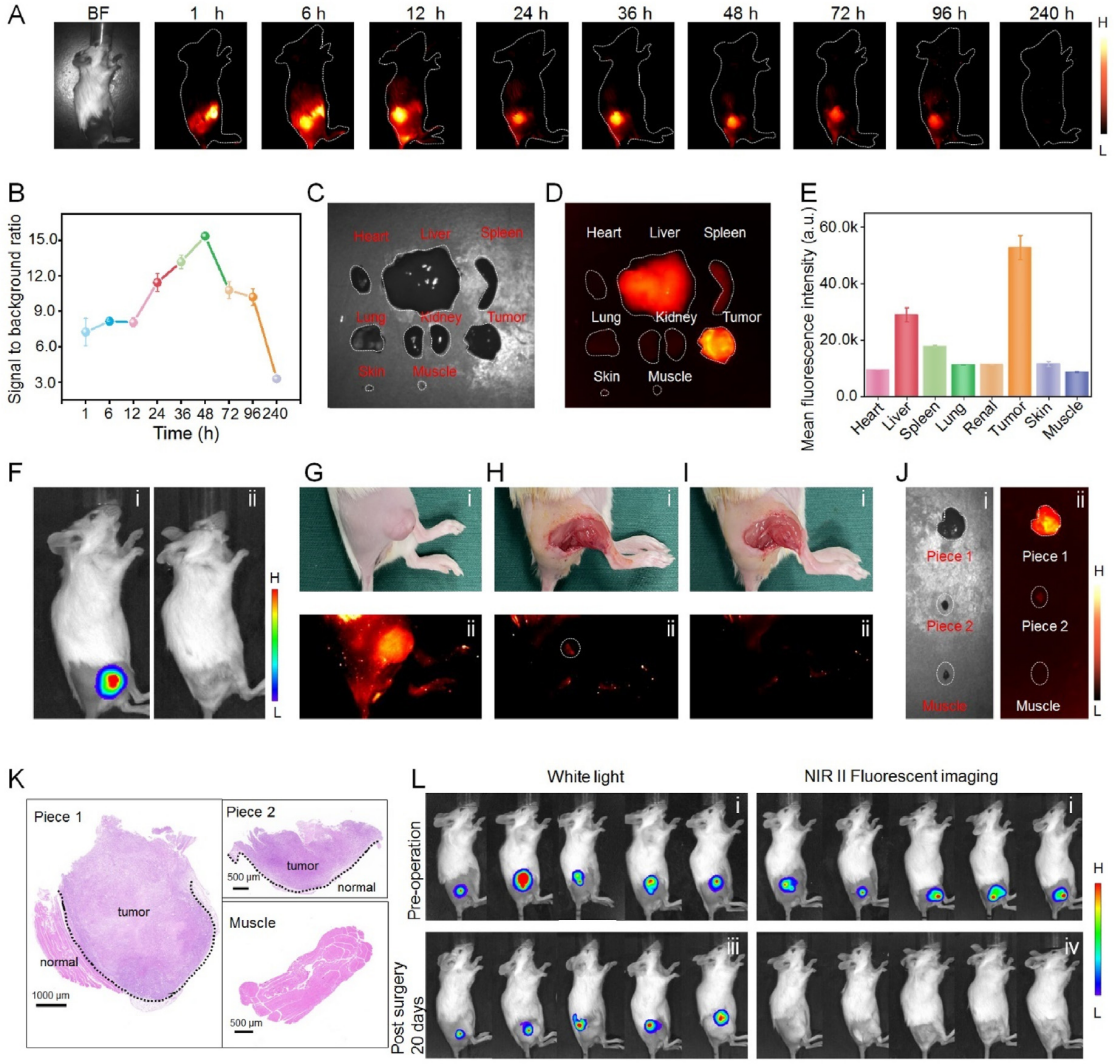
(A) Bright field image and NIR II fluorescence bioimages (1000 ​nm long-pass filter) of the liver tumor bearing mice after tail vein injection of DCNPs@Si-omSi-RGD for different timepoints. (B) Signal to background ratio (SBR) in NIR II fluorescence images from A. *Ex vivo* bright field (C) and NIR II fluorescent images (D) of main organs, tumor and normal tissues (skin and muscle) at 48 ​h post-injection. (E) Quantitative fluorescent intensity analysis in D. Bioluminescent images of liver tumor bearing mice before (i) and after NIR II fluorescent imaging guided tumor surgery (ii). Bright field (Gi) and NIR II fluorescent images (Gii) of subcutaneous liver tumor bearing mouse. Bright field (Hi) and NIR II fluorescent images (Hii) of first tumor resection. Bright field (Ii) and NIR II fluorescent images (Iii) of second residual tumor resection. Bright field (Ji) and NIR II fluorescent images (Jii) of dissected tumor tissues and peripheral muscle tissue. (K) H&E staining images of Piece 1, Piece 2 and surrounding muscle tissue in Piece 2. (L) Representative bioluminescence images of liver tumor bearing mice before and after 20-days of surgery. Tumors were removed under white light (left) or NIR II fluorescence imaging assistance (right).

Based on above tumor cell targeting capability of DCNPs@Si-omSi-RGD *in vitro*, tumor tissue precise discrimination capability was then evaluated. Firstly HepG2-Luc cells were intramuscularly inoculated into 6-week-old female Balb/c mice. When the tumor volume reached to ∼200 ​mm^3^, the nanoprobes (5 ​mg/kg) were then intravenously injected into the liver tumor-bearing mice. Secondly, non-invasive NIR II fluorescence imaging (808 ​nm laser, 400 ​mW/cm^2^, 1000 ​nm filter pass) was performed at particular time periods (Fig. 5A). Tumor tissue could be gradually demarcated from the surrounding normal tissues during 6–96 ​h post-injection, the tumor accumulation reached a maximum level at 12 ​h. Tumor outline could be clearly visualized at 48 ​h and hardly any substantial nonspecific fluorescent signals were observed from the major organs and muscle tissues surrounding the tumor. Additionally, peaking SBR (tumor to peritumoral muscle tissue) was 15.3 ​at 48 ​h post-injection (Fig. 5B), which was also consistent with the results of total biodegradation time point of DCNPs@Si-omSi under GSH stimuli described in Fig. 3A, suggesting the reliability of our nanoprobes for pinpointing location of malignant tumors. *Ex vivo* NIR II fluorescent imaging was further conducted at 48 ​h post-injection to assess the biodistribution of DCNPs@Si-omSi-RGD in major organs (Fig. 5C, **D**). The results showed that the fluorescent signal was mainly observed in tumor, in addition, only weak fluorescent intensity was found in RES and normal tissues (skin and muscle), unambiguously verifying the actively targeting to tumor and prominent NIR II signal recover capability of our nanoprobe under high GSH stimuli (Fig. 5E). Meanwhile, according to *ex vivo* fluorescent imaging at other timepoints, significantly higher tumor signal was also observed in 48 ​h post-injection group (Fig. S11, **S12**), in consideration of the optimal SBR at 48 ​h, all tumor surgical investigations should be performed at this time point. Subsequently, *in vivo* NIR II fluorescent imaging *via* the assistance of DCNPs@Si-omSi-RGD for tumor surgery navigation was studied. Firstly, the tumor profile was non-invasively evaluated by bioluminescence imaging *via* tail vein injection of D-luciferase potassium that is capable of specifically recognize HepG2-Luc cells (Fig. 5Fi). Secondly, 5 ​mg/kg DCNPs@Si-omSi-RGD was intravenously injected into mice, and the NIR II fluorescent imaging was carried out after 48 ​h tail vein administration (Fig. 5Gi, **Gii**). Interestingly, compared to the bioluminescent imaging, more institutive liver tumor outline could be obtained attributing to higher resolution and penetration depth of NIR II light. These imaging findings indicated that DCNPs@Si-omSi-RGD could be regarded as the NIR II contrast agent for intraoperatively identify malignant tumors. Immediately, to stimulate intraoperative tumor discrimination, the main solid tumor was removed under NIR II fluorescent imaging guidance (first cut, Piece 1). Upon removing the bulk of the solid tumor, we used NIR II fluorescent imaging system to examine any residual fluorescent signals associated with leftover tumor lesions. Importantly, a residual tumor tissue was unambiguously visualized with a well-defined delineation under NIR II fluorescent imaging system (Fig. 5Hi, **Hii**). We excised the NIR II emitting residual tumor l until no fluorescent signal was detected in the original tumor location (second cut, Piece 2) (Fig. 5Ii, **Iii**). Besides, no bioluminescent signal was detected in postoperative mice, preliminarily hinting the complete tumor surgery (Fig. 5Fii). Finally, Piece 1, Piece 2 with strong NIR II signal and peritumoral muscle tissue of Piece 2 with no NIR II signal (Fig. 5J) were further sliced to make thin sections for hematoxylin and eosin (H&E) staining. The boundaries between tumor tissue and normal tissue could be clearly found in both dissected tumor tissues and no residual tumor cells were detected in the muscle tissue (Fig. 5K). High-resolution imaging resolved small residual cancerous tissue (∼1 ​mm^3^) during surgery, allowing thorough and nonexcessive tumor removal. Furthermore, we also designed multiple parallel controlled trials for obtaining a comprehensive comparison of tumor recurrences/metastases after resection under the guidance of NIR II fluorescent imaging and white light. All subcutaneous tumors were preoperatively confirmed by bioluminescence imaging. No tumor recurrences/metastases occurred in the fluorescence imaging group after 20-day post-operation, while all mice underwent removal surgery under white light were detected bioluminescent signals (Fig. 5L). Statistical results showed that, in comparison with white light, significant suppression of tumor recurrence rate was found in NIR-II fluorescence imaging group (5.61% *vs* 95.61%, ∗∗p ​< ​0.01), further proved that our nanoprobes were considerably efficient for specifically labeling cancerous tissues during complete tumor surgery (Fig. S13).

### 3.5 Systematic toxicity evaluation

**Fig. 6:**
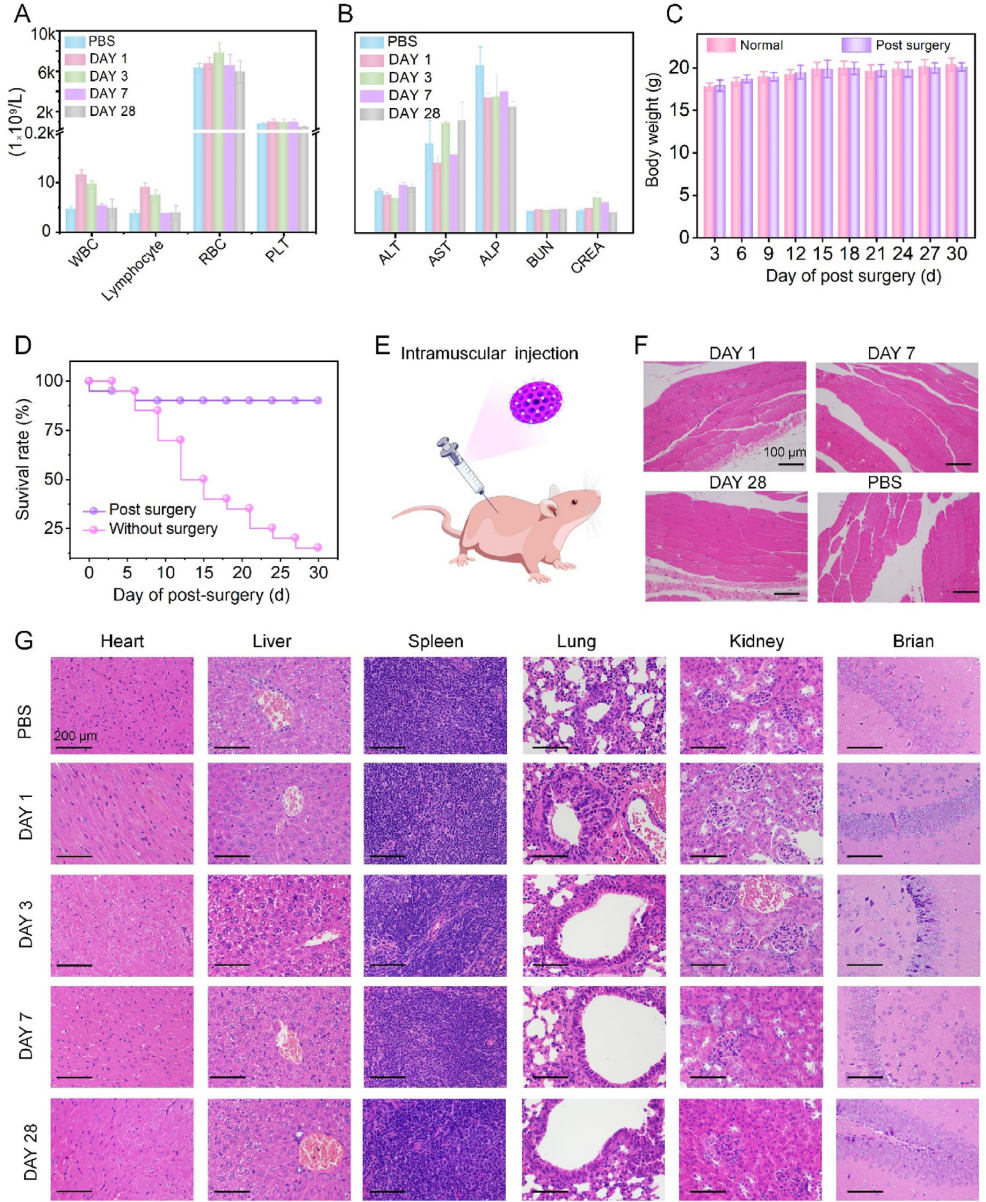
(A) Various biochemical indicators, (B) blood routine indexes, (C) body weight fluctuations and (D) survival rates of Balb/c mice after NIR II fluorescent imaging guided tumor surgery for different days. Schematic illustration of intramuscular injection of DCNPs@Si-omSi-RGD (E) and the corresponding H&E staining images of muscle after intramuscular injection of 1, 7, 28 days. Intramuscular injection of PBS for 1 day was set as the control group (F). (G) H&E staining results of the of heart, liver, spleen, lung, kidney and normal tissue (brain) from normal mice which were sacrificed at 1st, 3rd, 7th, 28th day after tail vein injection with NIR II nanoprobes. Tail vein injection of PBS for 1 day was set as the control.

Inspired by above tumor complete resection under NIR II fluorescence imaging navigation, we finally conducted the systematic toxicity studies of this NIR II contrast agent. Firstly, main hematological parameters, blood biochemical parameters, body weight and survival rates were surveilled after 28-day post-operation. The hematological results showed that compared with the PBS group, negligible fluctuations were found in lymphocyte, RBC and platelet of our nanoprobe. Only WBC increased at the 1st day 1 and 3rd day, but it completely returned to normal condition at the 7th day (Fig. 6A), indicating that slight inflammatory reaction occurred in a short time after the tumor surgery. Biochemical results presented that the corresponding functional factors in liver and kidney had no significant changes when compared with the control group. It proved that intravenously injected DCNPs@Si-omSi-RGD for tumor surgery had no adverse effects on the hepatic and renal functions (Fig. 6B). Furthermore, no abnormal body weight changes were found within 28-day post-operation in comparison with normal health mice (Fig. 6C). Concurrently, more than 90% mice survived in fluorescent imaging surgery, while only 10% mice were alive in white light assisted surgery at the 30th day (Fig. 6D). Apparently, all results suggested that our nanoprobes had good biocompatibility with no disturbance on health recovery and tumor surgery under NIR II fluorescence imaging guidance, and could also prolong the long-term survival rate. Subsequently, pathological staining experiments were carried out to observe the acute and long-term toxicity *via* intramuscular injection of our nanoprobes dispersed in PBS into normal Balb/c mice. The results showed that compared with PBS group, no obvious inflammatory responses occurred in muscle tissue even after at 1st, 3rd and 28th day of DCNPs@Si-omSi-RGD injection (Fig. 6E, **F**). Such a biocompatible and nontoxic properties of our NIR II nanoprobe is latterly verified by H&E assessments in major organs and normal tissues randomly harvested from Balb/c mice after intravenous injection of 5 ​mg/kg DCNPs@Si-omSi-RGD. Similar with PBS group, no marked damage can be detected in the heart, liver, spleen, lung, kidney and brain after 1- and 28-day treatments (Fig. 6G). As biosafety is always the major concerns in clinical applications, preventing off-target effects and exploring the appropriate long-term toxicities are of vital importance. As expect, all results proved that DCNPs@Si-omSi-RGD presented no obvious adverse effect on animals with tumor surgery and healthy mice holding a great clinical potential for translation in various malignant tumor resections.

## 4 Conclusions

In conclusion, we have synthesized Nd-based nanoprobes with a core-shell nanostructure which rendered the NIR II nanoprobes with tetrasulfide incorporation in the silica framework. After RGD modification, our DCNPs@Si-omSi-RGD could specifically binding to liver tumor cells to dramatically recover NIR II fluorescence under intracellular GSH triggering. This is mainly due to the decomposition of silica shells in tumor cells. Interestingly, our nanoprobes exhibited tumor precise targeting in subcutaneous liver tumor bearing Balb/c mice with significant NIR II fluorescence enhancement after 48 ​h of tail vein injection with DCNPs@Si-omSi-RGD. The fluorescence enhancement of our nanoprobes *in vivo* is consistent with the degradation trend of the silica shells *in vitro*, proving the reliability of our GSH responsive nanoprobe for boosting tumor margin determination. Tumors could be completely resected under NIR II fluorescence imaging navigation at this timepoint, confirming by bioluminescent imaging and H&E staining. Importantly, our NIR II nanoprobe showed no long-term toxic side effects with significant prolonging survival rate. In view of above results, our Nd-based nanoprobes hold advantages of outstanding photostability, high biocompatibility and ultrasensitive tumor microenvironment SBR improvement over other NIR II fluorescent probe like QDs, organic dyes and SWCNTs for tumor resection navigation, laying down a sound foundation for future translation of NIR II contrast agents.

## Data availability

All experimental data within the article are available from the corresponding author upon reasonable request.

## Declaration of competing interest

The authors declare that they have no known competing financial interests or personal relationships that could have appeared to influence the work reported in this paper.
