# Supplementary material for: Tumor microenvironment enhanced NIR II fluorescence imaging for tumor precise surgery navigation via tetrasulfide mesoporous silica-coated Nd-based rare-earth nanocrystals
Source: Mater Today Bio. 2022 Aug 20;16:100397. doi: 10.1016/j.mtbio.2022.100397 (PMC9445393; doi:10.1016/j.mtbio.2022.100397)
Supplement: Multimedia component 1 [file mmc1.docx]

Tumor Microenvironment Enhanced NIR II Fluorescence Imaging for Tumor Precise Surgery Navigation *via* Tetrasulfide Mesoporous Silica-coated Nd-based Rare-earth Nanocrystals

Jiaqi Li^1,2^, Fukai Zhu^4^, Kangliang Lou^5^, Haina Tian^2,6^, Qiang Luo^1,2^, Yongying Dang^5^, Xiaolong Liu^1,2,^*, Peiyuan Wang^1,2,^*, Liming Wu^1,3,^*

1. The United Innovation of Mengchao Hepatobiliary Technology Key Laboratory of Fujian Province, Mengchao Hepatobiliary Hospital of Fujian Medical University, Fuzhou 350025, P. R. China.
2. Key Laboratory of Design and Assembly of Functional Nanostructures, Fujian Institute of Research on the Structure of Matter, Chinese Academy of Sciences, Fuzhou 350002, P. R. China.
3. Department of Hepatobiliary and Pancreatic Surgery, The First Affiliated Hospital, Zhejiang Provincial Key Laboratory of Pancreatic Disease, School of Medicine, Zhejiang University, Hangzhou 310003, China
4. Collaborative Innovation Center of Mushroom Health Industry, Minnan Normal University, Zhangzhou, Fujian 363000, P. R. China.
5. Cancer Center & Department of Breast and Thyroid Surgery, Xiang’an Hospital of Xiamen University, School of Medicine, Xiamen University, Xiamen 361100, Fujian, China.
6. Department of Biomaterials, College of Materials, Research Center of Biomedical Engineering of Xiamen & Key Laboratory of Biomedical Engineering of Fujian Province, Xiamen University, Xiamen 361005, P. R. China

*Corresponding authors: liuxl@fjirsm.ac.cn, [wangpeiyuan@fjirsm.ac.cn](mailto:wangpeiyuan@fjirsm.ac.cn), wlm@zju.edu.cn

**Part A: Supplementary Experimental Section**

**1. Experimental Section**

***1.1. Materials***

Anhydrous yttrium (III) chloride (YCl_3_, 99.9%), anhydrous neodymium (III) chloride (NdCl_3_, 99.9%), NaOH (99.9%), NH_4_F (99.99%), Y(CH_3_COO)_3_﹒*x*H_2_O (99.9%) and sodium trifluoroacetate (Na-TFA, 97%), Hexadecyltrimethylammonium bromide (CTAB), tetraethyl orthosilicate (TEOS), bis[3-(triethoxysily)propyl] tetrasulfide (BTES), triethanolamine (TEA), were purchased from Aladdin Industrial Inc. 1-octadecene (ODE, 90%) and oleic acid (OA, 90%) were purchased from Sigma-Aldrich. Methanol, *n*-octanol, triton X-100, ammonia, cyclohexane, ethanol and chloroform were purchased from Beijing Chemical Reagents, China. 1,2-distearoyl-sn-glycero-3-phosphoethanolamine-*N*-amino (polyethylene glycol) (DSPE-PEG_2000_-NH_2_) was purchased from Shanghai Ponsure Biotech, Inc and cRGD was supplied by GL Biochem (Shanghai) Ltd.

***1.2. Instruments***

Transmission electron microscopy (TEM) measurements were carried out on a JEM 2100F microscope (Japan) operated at 100 kV. Scanning electron microscope (SEM) measurement was analyzed using Nanoscope V multimode atomic force microscope. The luminescence spectra of all the samples were measured on an Edinburgh FLS-980 fluorescence spectrometer. Dynamic light scattering (DLS) and Zeta-potential were obtained on a NanoBrook Omni. UV–vis–NIR absorption spectra were measured on a Shimadz spectrophotometer (UV-3150) (Japan) with wavelength range of 300-1200 nm, unless otherwise specified, all spectra were collected under identical experimental condition. Confocal laser scanning microscope images were acquired in Nikon A1RMP imaging system. In vivo NIR II fluorescent images were obtained under a NIR-OPTICS Series III 900/1700 system (808 nm laser irradiation with a 1000 nm long filter pass).

***1.3. Synthesis of Nd-based rare-earth core-shell nanocrystals NaYF_4_: 5% Nd@NaYF_4_.***

*Synthesis of NaYF_4_: 5% Nd*: A mixture of anhydrous YCl_3_ 0.185g (0.95mmol), anhydrous NdCl_3_ 0.0125g (0.05mmol), OA (6 mL), and ODE (15 mL) was added to a 100 mL three-necked flask. Then, the mixture was heated to 140°C for 30 min under a vacuum environment. After the powder dissolved completely, the mixture was cooled to room temperature, then 10 mL of a methanol solution containing 4 mmol of NH_4_F (0.1482g) and 2.5 mmol of NaOH (0.1g) were added. The reaction was then heated to 90°C with 30 min maintaining to remove all methanol. Subsequently, the reaction was heated to 300°C and kept with argon gas atmosphere for 60 min. After the solution cooled to room temperature, the product was collected by centrifugation (8000 rpm) with adding excess ethanol for more than 3 times, finally, the core sample was dispersed in 10 mL of cyclohexane and stored at 4°C for further coating.

*Synthesis of Y-OA precursor*: A mixture of Y(CH_3_COO)_3_﹒*x*H_2_O 0.6651g (2.5mmol), OA (10 mL), and ODE (15 mL) was added to a 100 mL three-necked flask. Then it was heated to 140°C for 60 min under a vacuum, finally, the transparent Y-OA precursor (0.1 M) was obtained.

*Synthesis of Na-TFA-OA precursor*: A mixture of Na-TFA (4 mmol) and OA (10 mL) was added to a 50 mL three-necked flask. The mixture was then stirred at room temperature and pumped with a vacuum for 6 h, and then the transparent Na-TFA-OA precursor (0.4 M) was obtained.

*Synthesis of NaYF_4_:5%Nd@NaYF_4_ core-shell nanocrystals*: The core-shell nanocrystals were synthesized by the successive layer-by-layer (SILAR) strategy. A mixture of NaYF_4_: 5% Nd (0.5 mmol), OA (8 mL) and ODE (12 mL) was added to a 100 mL three-necked flask. Then, it was heated to 80°C for 30 min under a vacuum to remove cyclohexane. Latterly, the reaction was heated to 300°C under argon atmosphere with the alternate injection of Y-OA precursor (1 mL) and Na-TFA-OA precursor (0.5 mL) every 15 minutes. After 16 pairs of injections, the reaction solution was cooled to room temperature. The final product was washed by ethanol and dispersed in 10 mL of cyclohexane.

*Synthesis of* *DCNPs@Si-omSi-PEG*: The DCNPs@Si-omSi (0.1 mmol) and DSPE-PEG_2000_-NH_2_ (20 mg) were dissolved in chloroform (5 mL). Then, amino lipid modified DCNP@Si-omSi (DCNP@Si-omSi-PEG) was acquired after slow evaporation of all of the chloroform in a fume cupboard. After removing excess DSPE-PEG_2000_-NH_2_ by ultra-centrifugation (12,000 rpm), the as-prepared DCNP@Si-omSi-PEG was obtained and re-dispersed in deionized (DI) water.

*Synthesis of* *DCNP@Si-omSi-RGD*: 5 mg of RGD was firstly dissolved in 2-(*N*-Morpholino) ethanesulfonic acid (MES) solution (5 mL, 0.1 M, pH 5.5), and then *N*-(3-Dimethylaminopropyl)-*N*′- ethylcarbodiimide hydrochloride (EDC, 20 mg), *N*-Hydroxysuccinimide (NHS, 20 mg) were added and stirred gently for 2 h. Subsequently, DCNP@Si-omSi-PEG (0.1 mmol) were added into the above solution under continuously stirring for 12 h. The RGD functionalized DCNP@Si-omSi was washed by DI water (12,000 rpm). The final sample, DCNP@Si-omSi-RGD was dispersed in DI water.

***1.4. Cellular toxicity assessment.***

*Cell culture*: HepG2 cells were obtained from the American Type Culture Collection (ATCC Manassas, VA, USA), HepG2-Luc cells were obtained from the Shanghai Zhong Qiao Xin Zhou Biotechnology Co., Ltd., China. Annexin V-FITC/PI apoptosis Kit was purchased from Beyotime Biotechnology. HepG2 cells and HepG2-Luc cells were cultured in DMEM medium containing 10% FBS, 1% penicillin and streptomycin. All cells were cultured in a standard humidified cell culture incubator (37°C, 5% CO_2_).

*Cell viability*: When the HepG2 cell density was reached about 90%, they were digested and re-dispersed evenly in 96 well plates (1000 cells per well). After 12 h of incubation, the cells were treated with DCNP@Si-omSi-RGD at different concentrations (25 μg/mL, 50 μg/mL, 100 μg/mL, 150 μg/mL, 200 μg/mL, 250 μg/mL, 300 μg/mL, 350 μg/mL, 400 μg/mL) for 12 h. The DMEM medium was then removed and the cells were washed with sterile PBS twice, then the pre-prepared 100 μL fresh DMEM medium with 0.5 mg/mL MTT was added for 2-4 h incubation under dark condition. 100 μL DMSO was added and the absorbance value at the wavelength of 492 nm was measured by a microplate reader.

*Cell apoptosis/necrosis*: Firstly, DCNP@Si-omSi-RGD (PBS, 25 μg/mL, 100 μg/mL, 200 μg/mL, 300 μg/mL and 400 μg/mL) treated HepG2 cells were digested with trypsin and washed with PBS. Then all cells were suspended in aqueous mixture with 195 μL Annnexin V-FITC binding solution, 5 μL Annexin V-FITC and 10 μL propidium iodide staining solution. Finally, all cells were incubated at room temperature for 15 min and were further analyzed by a flow cytometer.

***1.5. NIR II fluorescent imaging guided tumor surgery***

*Tumor targeting of DCNP@Si-omSi-RGD in subcutaneous mice models:* Balb/c mice bearing subcutaneous HepG2-Luc tumor were intravenously injected with DCNPs@Si-omSi-RGD. NIR II fluorescence images were obtained at the different time points (1 h, 6 h, 12 h, 24 h, 36 h, 48 h, 72 h, 96 h, 240 h) under 808 nm laser irradiation with 1000 nm long-path filter. The SBR of the tumors was obtained by the PSL Viewer software.

*Biodistribution of DCNPs@Si-omSi-RGD*: Subcutaneous tumor-bearing Balb/c mice (n = 5) were intravenously injected with DCNPs@Si-omSi-RGD, and then they were sacrificed at different times (1 h, 12 h, 24 h, 48 h, 72 h and 96 h) post-injection. Subsequently, major organs (heart, liver, spleen, lung and kidney), tumors and normal tissues (muscle, skin) were dissected and collected. Subsequently, the *ex vivo* NIR-II fluorescence images were acquired and the corresponding mean fluorescence intensity of these tissues was quantitatively analyzed to evaluate the biodistribution of DCNPs@Si-omSi-RGD.

*Blood routine and biochemical blood evaluation*: Balb/c mice after NIR II fluorescent tumor surgery were divided into five groups (n = 5): blank control group, 1^st^ day, 3^rd^ day, 7^th^ day, and 28^th^ day post-surgery groups. On the last day, blood samples were collected from each mouse for biochemical and blood routine evaluation, respectively. The blood samples for routine blood evolution were added with anticoagulant in advance and stored at 4°C. Afterwards, the blood samples were putted in water bath at 37°C for 2 h, followed by centrifugation at 1000 g for 5 min, and the upper serum was collected for biochemical blood evaluation.

**Part B: Supplementary Figures**

**
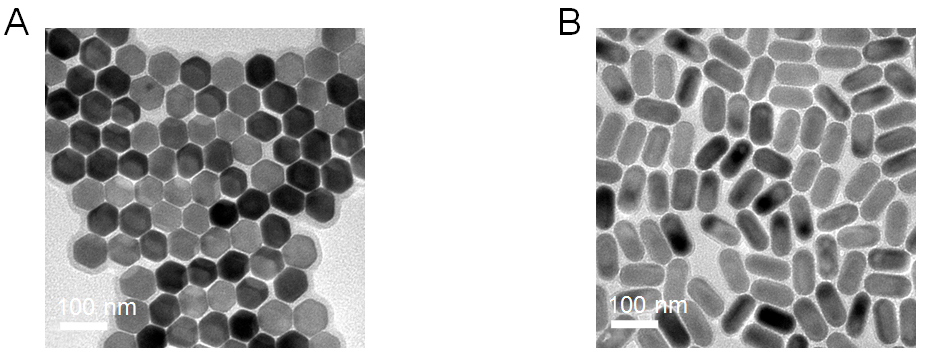
**

**Fig. S1.** TEM images of NaYF_4_:5%@ NaYF_4_ in *α*-phase (A) and *β*-phase (B).

**
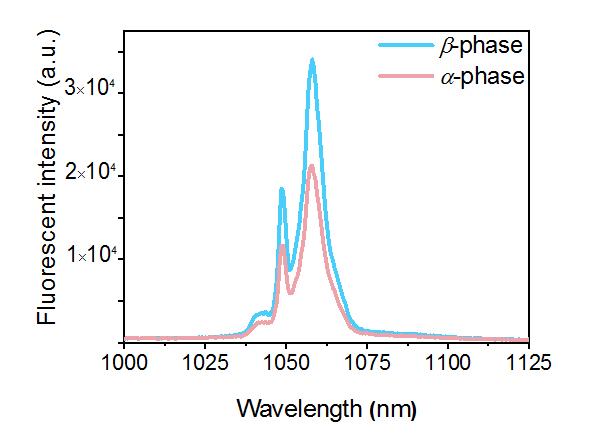
**

**Fig. S2.** Fluorescent emission spectra of NaYF_4_:5%@ NaYF_4_ in *α*-phase and *β*-phase under 808 nm laser irradiation.


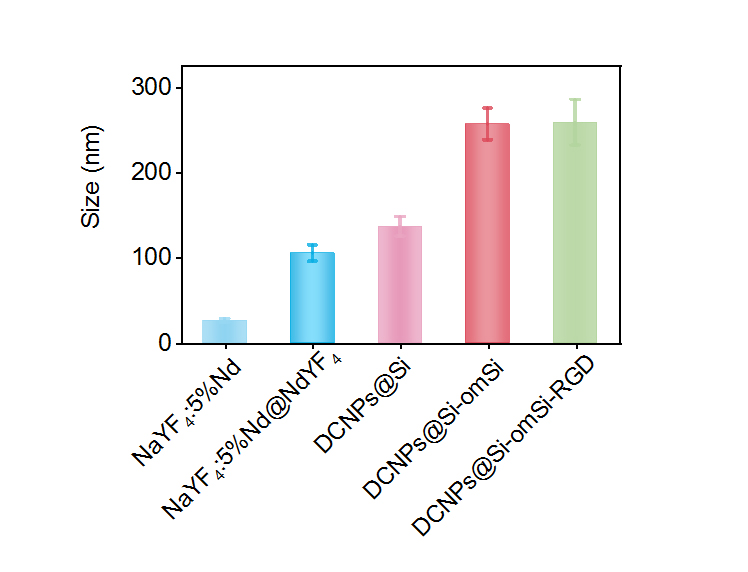


**Fig. S3.** The hydrodynamic diameter of NaYF_4_:5%, NaYF_4_:5%@ NaYF_4_, DCNPs@Si, DCNPs@Si-omSi and DCNPs@ Si-omSi-RGD.


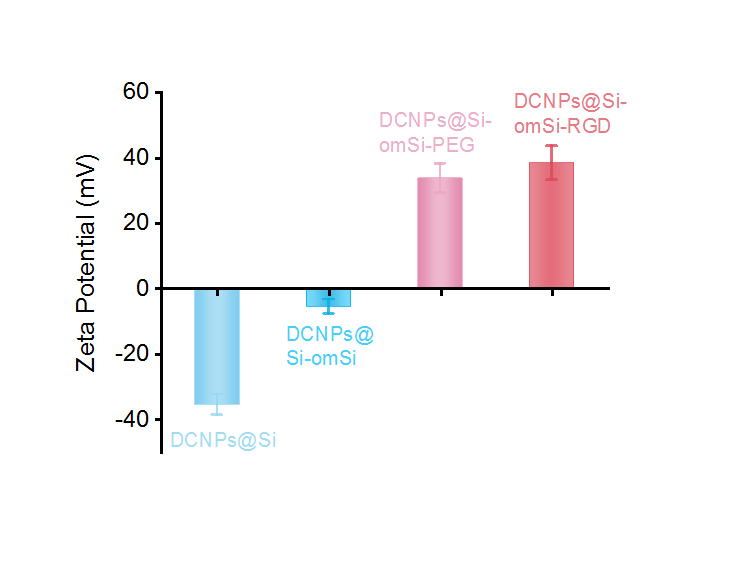


**Fig. S4.** The zeta potential values of DCNPs@Si, DCNPs@Si-omSi, DCNPs@Si-omSi-PEG and DCNPs@ Si-omSi-RGD in DI water at pH 7.0.


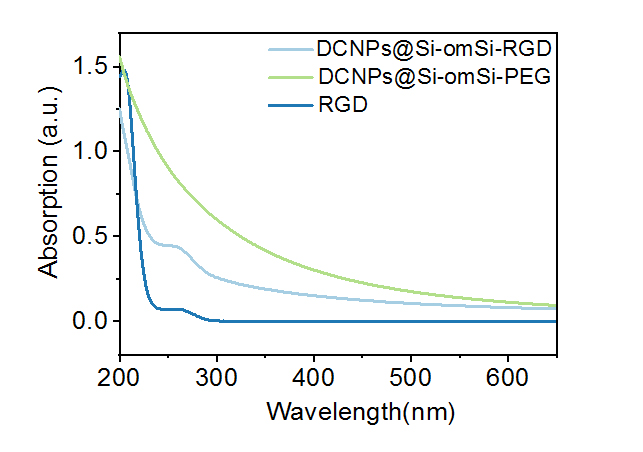


**Fig. S5.** The absorption spectra of free RGD, DCNPs@Si-omSi-PEG and DCNPs@ Si-omSi-RGD in DI water at pH 7.0.


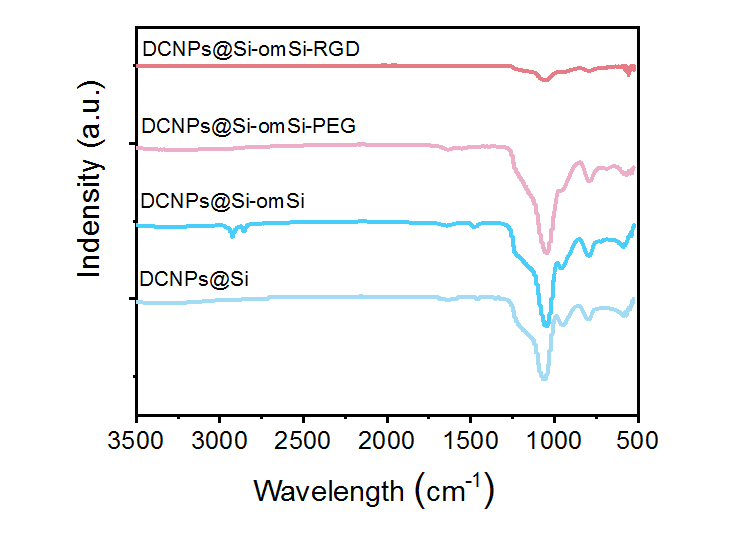


**Fig. S6.** FTIR spectrum of DCNPs@Si, DCNPs@Si-omSi, DCNPs@Si-omSi-PEG and DCNPs@ Si-omSi-RGD.


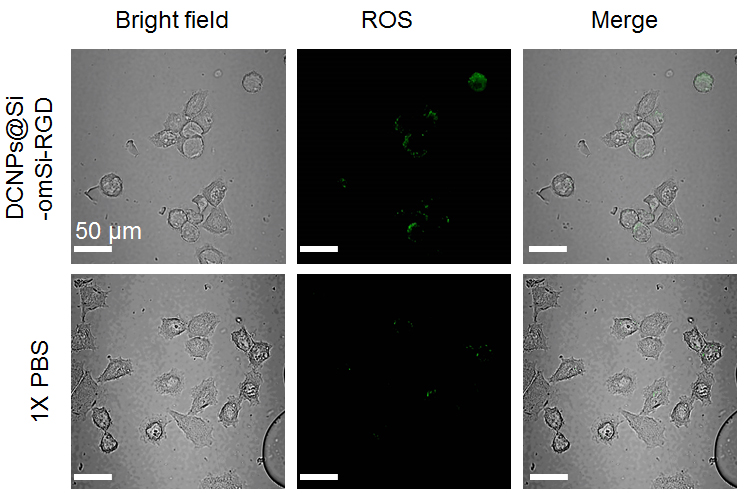


**Fig. S7.** ROS generation in HepG2 cells after incubated with 400 μg/mL DCNPs@Si-omSi-RGD for 12 h. Cells with PBS treatment was set as the control group.


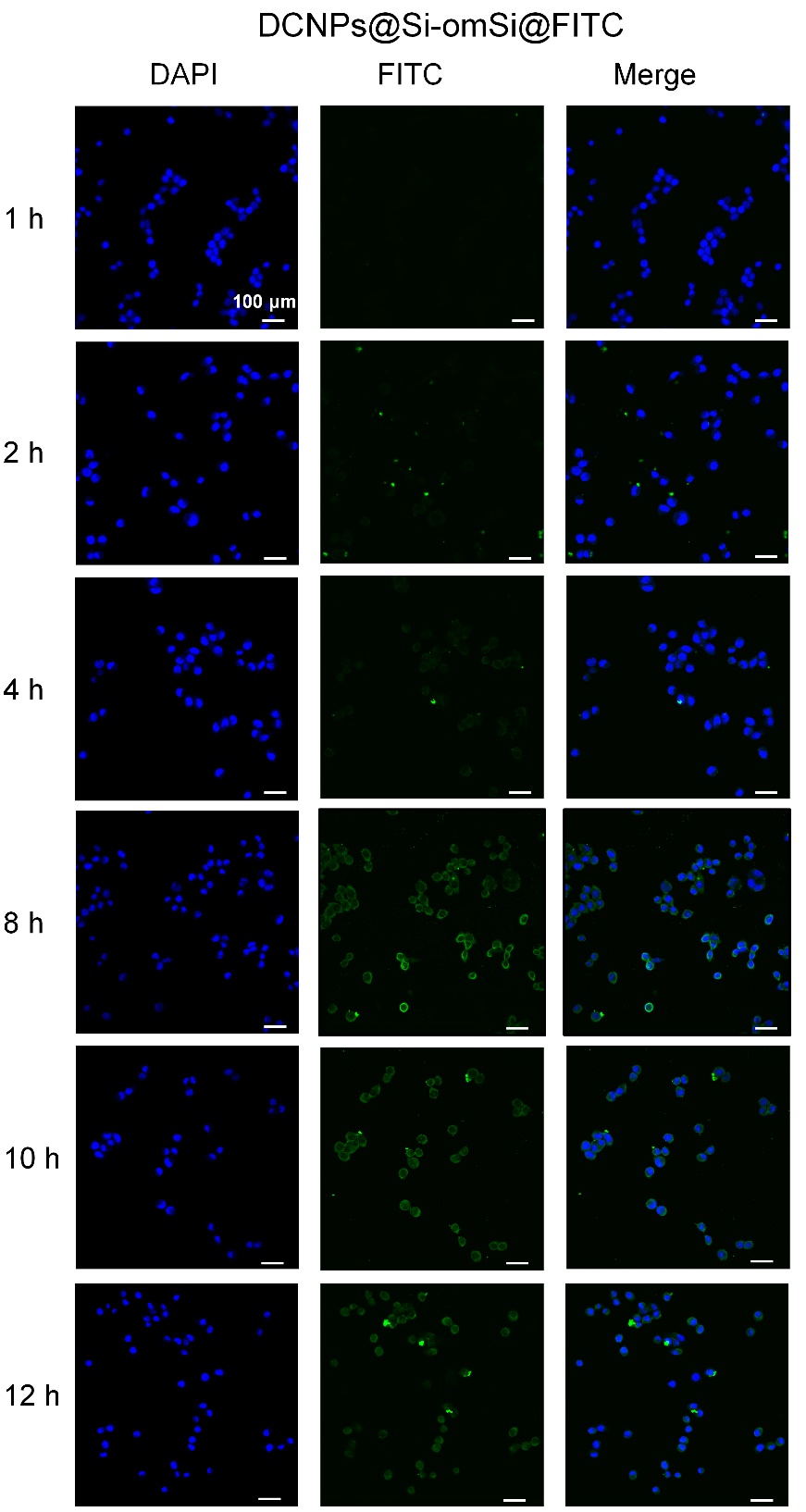


**Fig. S8.** CLSM images of HepG2 cells after treated with DCNPs@Si-omSi@FITC for different time points (1 h, 2 h, 4 h, 8 h, 10 h and 12 h).


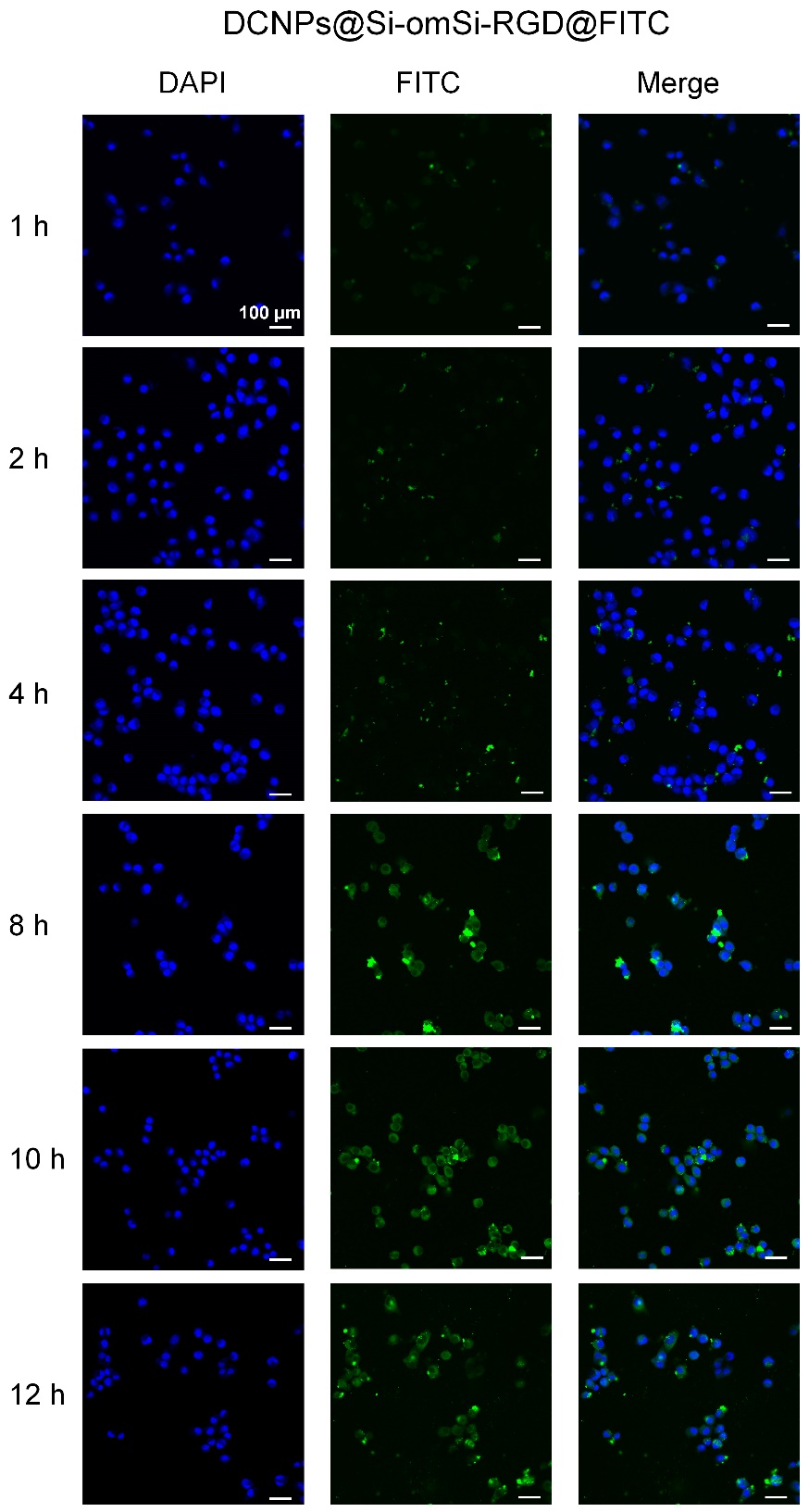


**Fig. S9.** CLSM images of HepG2 cells after treated with DCNPs@Si-omSi-RGD@FITC for different time points (1 h, 2 h, 4 h, 8 h, 10 h and 12 h).


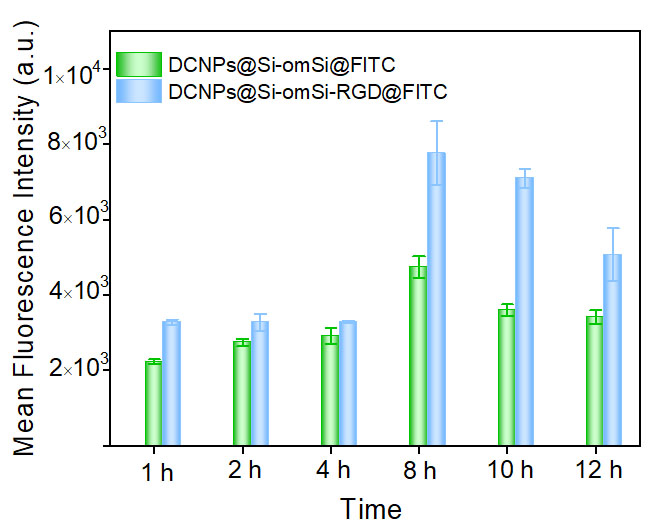


**Fig. S10.** Statistical analysis of the mean fluorescence intensity of cell uptake with DCNPs@Si-omSi@FITC and DCNPs@Si-omSi-RGD@FITC as the function of incubation time.


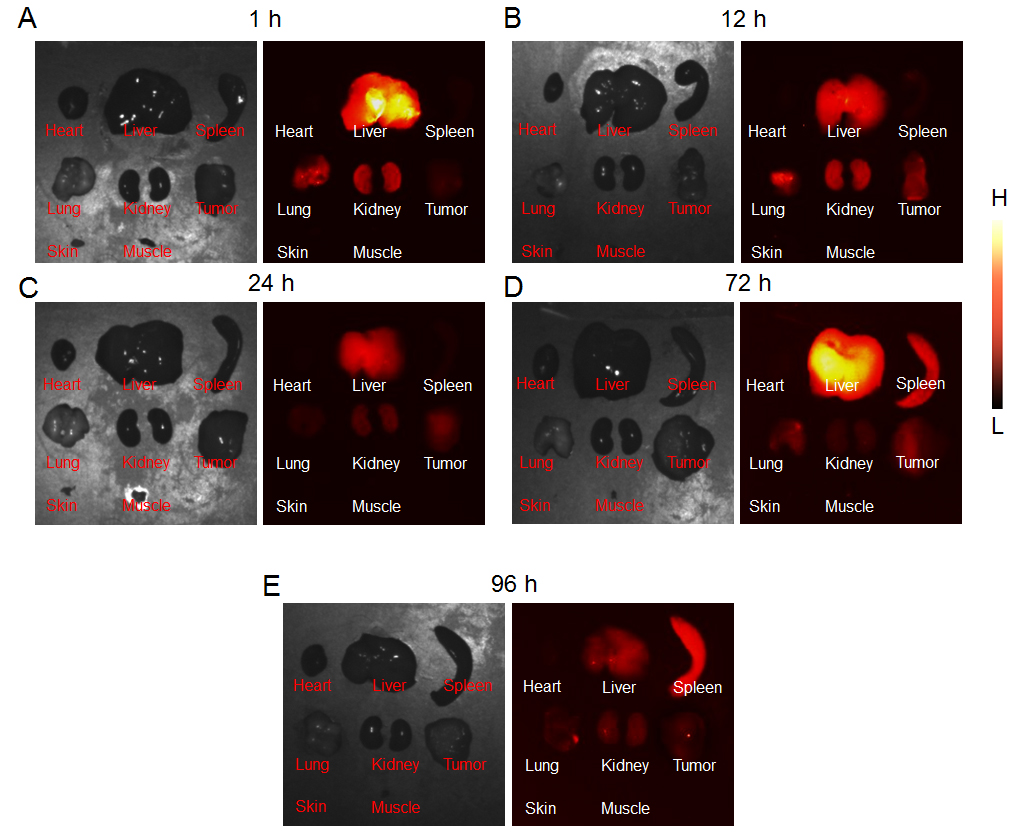


**Fig. S11.** *Ex vivo* NIR II fluorescence imaging of major organs (heart, liver, spleen, lung, kidney), tumor and normal tissues (skin and muscle) after dissection at 1 h, 12 h, 24 h, 72 h and 96 h post-injection of DCNPs@Si-omSi-RGD.


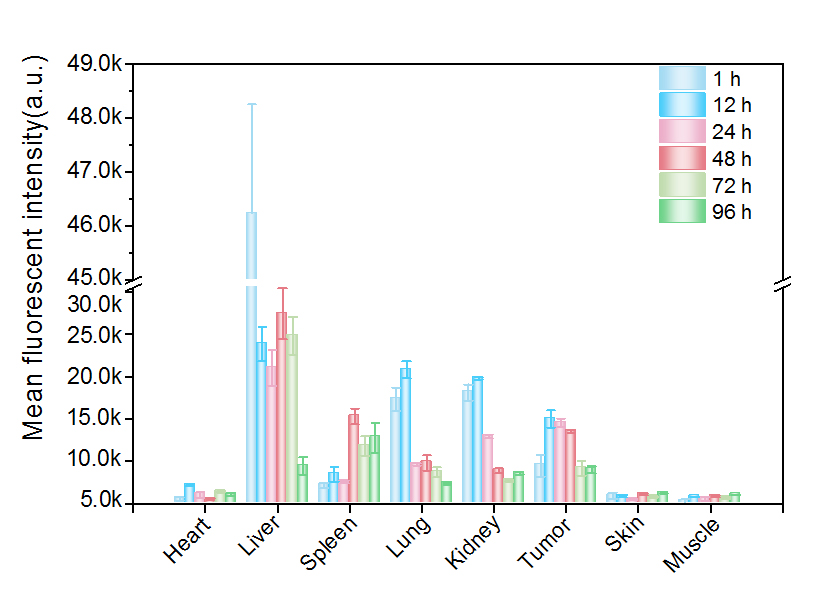


**Fig. S12.** Quantitative fluorescence intensity analysis in Figure S11.


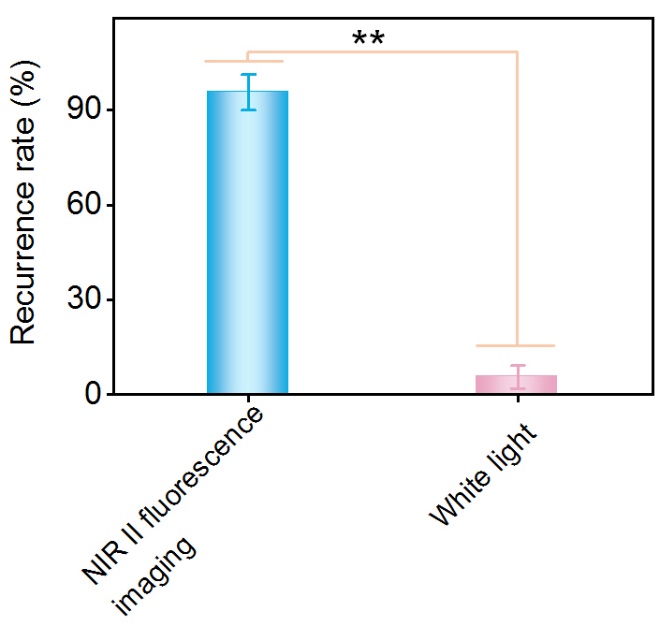


**Fig. S13.** Statistical analysis of tumor recurrence rate after tumor surgical resection under NIR II fluorescence imaging guidance or white light.
